# Personalized machine learning–based prognostic model for ICU-acquired bloodstream infections

**DOI:** 10.3389/fcimb.2025.1636886

**Published:** 2025-10-29

**Authors:** Shijun Zhou, Xilei Cai, Xiujuan Yang, Chuanchang Wu, Guomei Xia, Long Yu, Wanjun Liu, Zhenhua Zhang

**Affiliations:** ^1^ Department of Infectious Diseases, The Second Affiliated Hospital of Anhui Medical University, Hefei, China; ^2^ Institute of Clinical Virology, The Second Affiliated Hospital of Anhui Medical University, Hefei, China

**Keywords:** bloodstream infection, intensive care unit, prognostic model, machine learning, cross-validation, XGBoost, SHAP

## Abstract

**Background:**

Intensive care unit–acquired bloodstream infections (ICU-BSIs) are among the most prevalent healthcare-associated infections and a major cause of mortality among ICU patients. We developed a machine learning (ML)–based model to predict the prognosis of ICU-BSIs.

**Methods:**

Adult patients with blood cultures drawn ≥48 hours after ICU admission were included: the Second Affiliated Hospital of Anhui Medical University (AMU, China) and the Medical Information Mart for Intensive Care IV (MIMIC-IV, USA). The AMU dataset was used for model training and internal validation, and the MIMIC-IV dataset served as the external validation set. The model incorporated routinely collected, easily obtainable clinical variables, including several representing the average rate of change in laboratory indicators. After comparing multiple algorithms, eXtreme Gradient Boosting (XGBoost) was selected and optimized using cross-validation and grid search.

**Results:**

A total of 1,903 patients from AMU and 3,496 from MIMIC-IV were included. In both cohorts, antibiotic duration, platelet count, serum creatinine, duration of invasive mechanical ventilation, and Charlson Comorbidity Index (CCI) were significantly associated with 28-day mortality (*P* < 0.001). The XGBoost model using 33 variables showed strong discrimination, with an AUROC of 0.92 (95% CI 0.90–0.94) for training and 0.85 (95% CI 0.80–0.90) for internal validation. Shapley Additive Explanations (SHAP) identified the 10 most important variables; a simplified model using these maintained good accuracy, with AUROC values of 0.81 (95% CI 0.76–0.85) and 0.71 (95% CI 0.70–0.73) for the internal and external validation sets, respectively. In pathogen subgroups, the internal AUROC was 0.91 (95% CI 0.87–0.94) and 0.90 (95% CI 0.86–0.93) for Gram-positive (Gram+) and Gram-negative (Gram−) infections, with external validation AUROCs of 0.72 (95% CI 0.66–0.77) and 0.72 (95% CI 0.62–0.82), respectively.

**Conclusions:**

We developed and externally validated a personalized ML-based prognostic model for ICU-BSIs using multicenter time-series data. This model may facilitate early identification of high-risk patients, enabling timely intervention and optimized ICU resource allocation.

## 1 Introduction

Intensive care unit–acquired bloodstream infections (ICU-BSIs) represent one of the most frequent healthcare-associated infections in the intensive care unit (ICU) (Blot et al., 2022; Tabah et al., 2023; Vincent et al., 2020), with microbiologically confirmed incidence rates ranging from 3% to 7% (Ding et al., 2009; Garrouste-Orgeas et al., 2006; Massart et al., 2021; Ong et al., 2015; Thompson, 2008). These infections are associated with case fatality rates of 35%–40% (Buetti et al., 2024; Massart et al., 2021; Tabah et al., 2023), increase excess mortality among critically ill patients (Adrie et al., 2017; Garrouste-Orgeas et al., 2006; Massart et al., 2021; Prowle et al., 2011), and contribute to a considerable socioeconomic burden (Lambert et al., 2011; Laupland et al., 2006; Vandijck et al., 2008). Early identification and prompt intervention for high-risk patients can improve outcomes (Adrie et al., 2017; Loiodice et al., 2024; Seymour et al., 2017; Tabah et al., 2023).

Clinical scoring systems, which are frequently used for prognostic assessment and depend on static data, are unable to dynamically reflect changes in patient condition (Li et al., 2022; Minne et al., 2008). Furthermore, predicting the prognosis of ICU-BSIs based on pathogen data is limited by prolonged blood culture turnaround times and low positivity rates (Schwab et al., 2018). Although established statistical models are widely used for prognosis prediction, they are constrained by difficulty in capturing complex nonlinear relationships, reliance on specific distributional assumptions, and limitations in handling high-dimensional data and extensive interaction effects (Li et al., 2023; Russell et al., 2023; Wang et al., 2023).

Machine-learning techniques offer promising alternatives by overcoming these limitations through the integration of multidimensional predictors and capturing of complex nonlinear relationships (Ghafariasl et al., 2025; Lai et al., 2023). While these methods demonstrate notable strengths in the early detection, heterogeneity analysis, and prognostic assessment of sepsis (Hou et al., 2020; Rahman et al., 2024; Zhang et al., 2020), their generalizability is often limited by a narrow population or a lack of external validation. Therefore, there is a need for prognostic models targeting ICU-BSIs.

In this study, we aimed to develop a machine learning–based model for time-resolved prediction of 28-day mortality in patients with ICU-BSIs, which could enable the identification of high-risk populations and provide precise decision support for intensive care physicians.

## 2 Materials and methods

### 2.1 Data sources

This retrospective cohort study analyzed data from the Second Affiliated Hospital of Anhui Medical University (AMU) and the Medical Information Mart for Intensive Care IV (MIMIC-IV, version 3.0). The AMU database comprises records from a large regional medical center, whereas MIMIC-IV is a widely used, open-access database comprising data collected at the Beth Israel Deaconess Medical Center. Ethical approval was obtained from AMU (approval code YX2024-204) and access to MIMIC-IV was granted after completion of the required training (record 39691989). All patient data were de-identified and encrypted to ensure privacy, and the ethical documentation is available online. This study adheres to the Transparent Reporting of a multivariable prediction model for Individual Prognosis Or Diagnosis–Artificial Intelligence (TRIPOD-AI) reporting guidelines, and the completed checklist is provided in the **Supplementary Material**.

### 2.2 Patients and definitions

ICU-BSIs were diagnosed according to Centers for Disease Control and Prevention (CDC)/National Healthcare Safety Network (NHSN) guidelines (Horan et al., 2008). The inclusion criteria for suspected ICU-BSIs were: (a) blood cultures obtained ≥48 hours after ICU admission; (b) first ICU admission; and (c) age ≥18 years. The exclusion criteria were: (a) the presence of a related infection at another site; and (b) prior or repeated ICU admissions.

### 2.3 Data preprocessing

**Figure 1:**
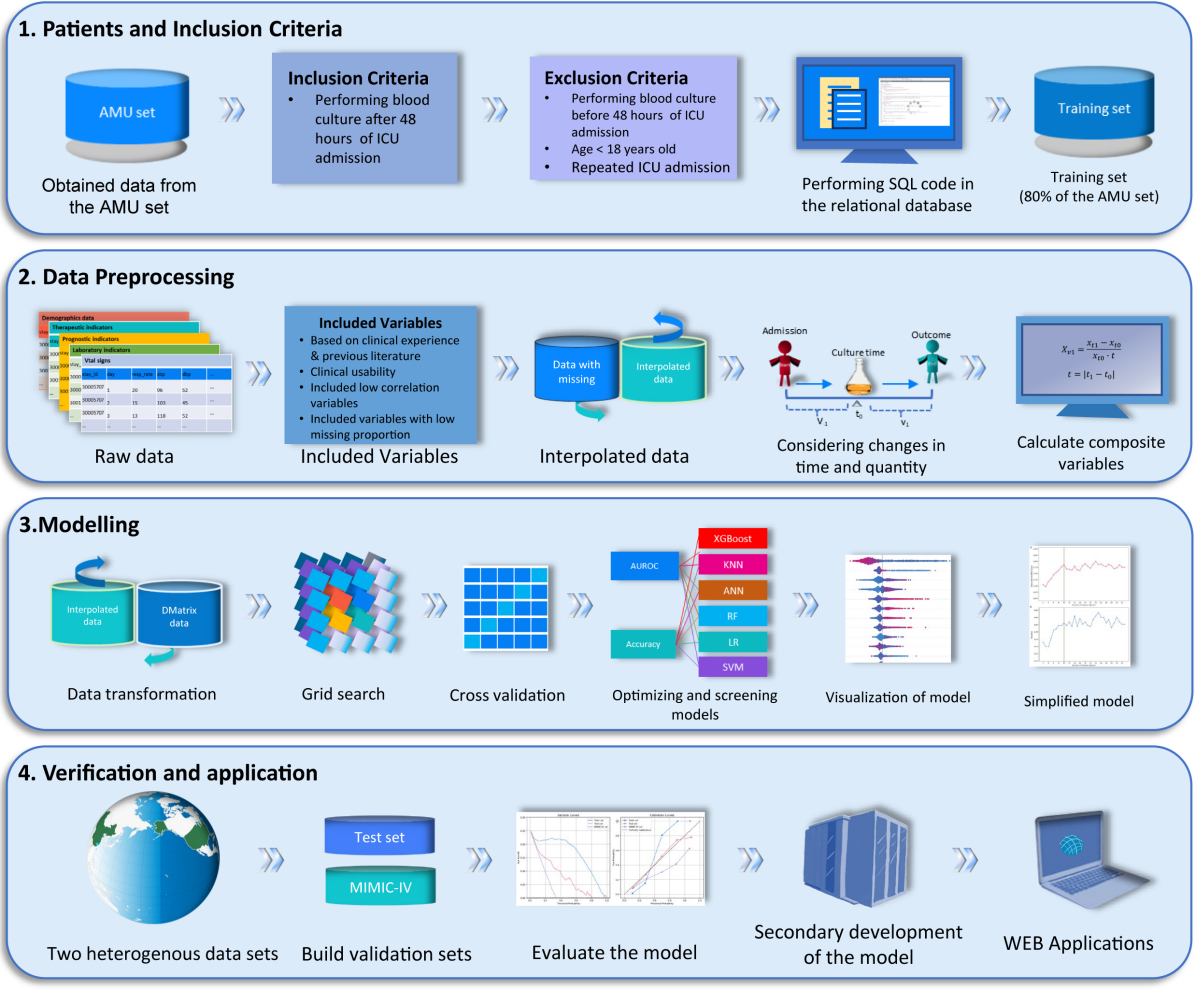
Study design. AMU, Anhui Medical University; ICU, Intensive Care Unit; MIMIC-IV, Medical Information Mart for Intensive Care IV.

**Figure 1** shows the study workflow. We included 5,399 patients: 3,496 from the MIMIC-IV dataset and 1,903 from the AMU dataset. AMU patients were randomly split into training and test sets (hereafter referred to as the internal validation set) in a 4:1 ratio. The training set was used for model development, whereas the test set and the MIMIC-IV dataset served as the internal and external validation sets, respectively. The model incorporated demographic factors, the Charlson Comorbidity Index (CCI), laboratory measurements, and treatment-related variables; the outcome was 28-day mortality (**Supplementary Table S1**). Based on established criteria for sample-size calculation (Riley et al., 2019), the sample size was adequate (**Supplementary Table S2**).

Variable selection was guided by clinical value and accessibility, acknowledging that ideal predictors should support early diagnosis and prognostication with adequate specificity and sensitivity. Variables were selected according to the following principles: (1) to maintain model parsimony, pairwise associations were minimized (absolute Spearman’s ρ < 0.5; **Supplementary Figure S1**); (2) variables with >50% missingness were excluded (**Supplementary Figure S2**); and (3) to ensure model stability and practicality, variables were chosen based on their clinical relevance, objectivity, and accessibility. Because this was a retrospective analysis, outliers and missing values were addressed before model development. For variables with <50% missingness, multiple imputation was performed using the Multivariate Imputation by Chained Equations (MICE) framework with m = 50 datasets: Predictive Mean Matching for continuous variables, logistic regression (LR) for binary variables, and polytomous (multinomial) regression for categorical variables; a fixed random seed (123) was used to ensure reproducibility. Analyses across imputed datasets were pooled using Rubin’s rules. This approach reduced bias, preserved statistical power, and enhanced model stability.

Given that patients’ laboratory parameters can exhibit substantial short-term fluctuations in patients with ICU-BSIs, variables were time-aligned to the blood culture collection timestamp, and change-related features were engineered to enhance patient-level personalization and reduce redundancy. In this study, *t_0_* denotes the blood culture collection time, serving as the baseline reference point, while *t_1_* represents the time of variable extraction, which may occur either before or after *t_0_*. Accordingly, when *t_1_* > *t_0_*, it indicates the post–blood culture period, and when *t_1_* < *t_0_*, it corresponds to the pre–blood culture period. The mean relative rate of change of a variable *X* between the two time points is defined as:


$$X_{v1}=\frac{x_{t1}-x_{t0}}{x_{t0}\cdot |\Delta t|}$$

Note: Δt=*t*_1_-*t*_0_

Based on this definition: *X_v1_* denotes the mean relative rate of change during the post–blood culture period (*t_1_* > *t_0_*); *X_v-1_* denotes the mean relative rate of change during the pre–blood culture period (*t_1_* < *t_0_*). *X_v-1_* represents the normalized rate of change rather than the maximum absolute value. Since the calculation produces multiple rates of change over time due to temporal rolling, we selected the rate with the largest absolute value. In this framework, positive values indicate an increase in the corresponding indicator, whereas negative values indicate a decrease.

### 2.4 Outcome definition

The follow-up period was defined as 28 days from the time of blood culture collection. Patients who remained alive at 28 days were classified as survivors. Data beyond the 28-day period were not incorporated into the model, and subsequent survival status was not further tracked.

### 2.5 Data analysis

**Figure 2:**
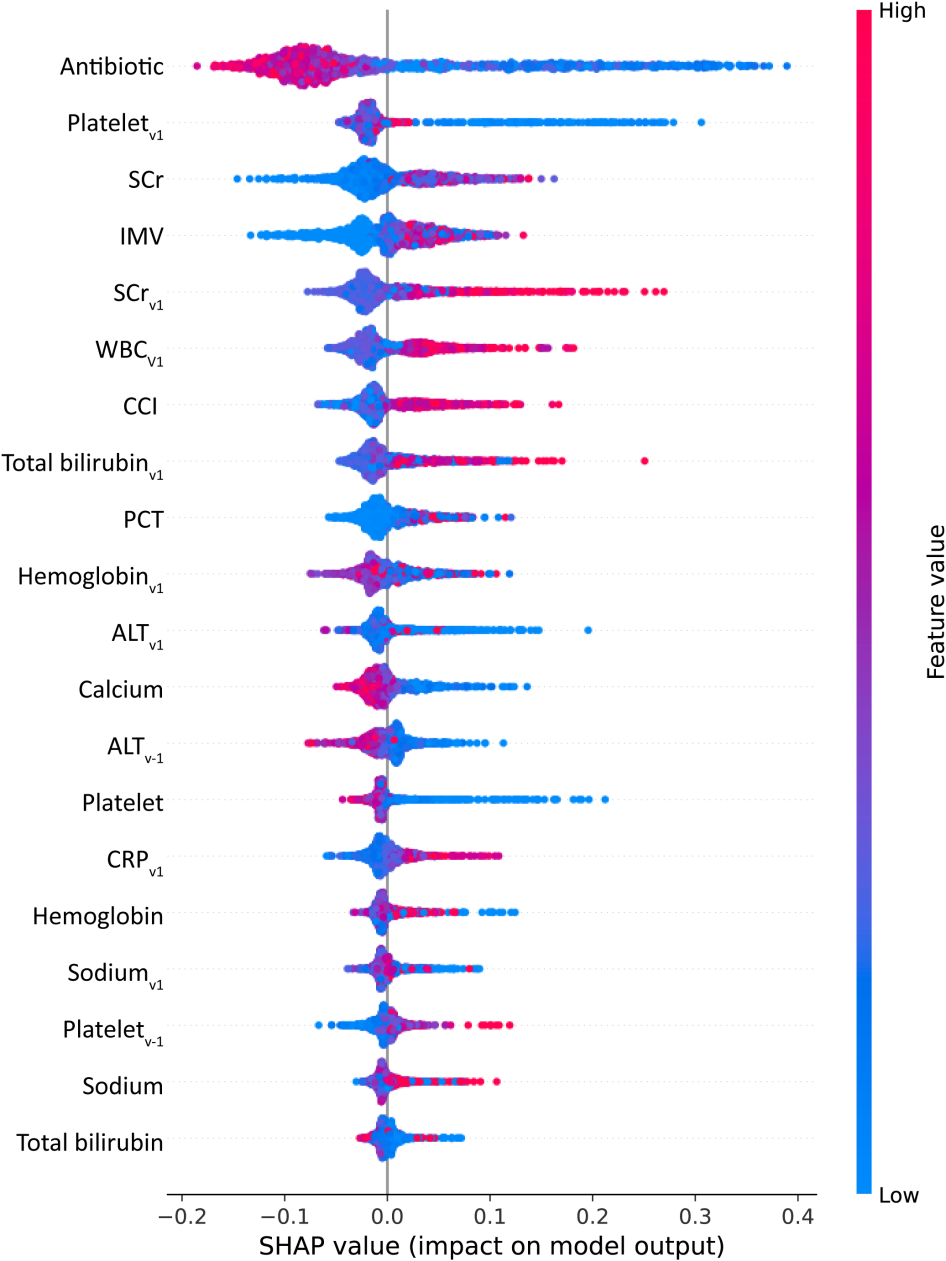
SHAP value rankings of variable contributions to 28-day mortality. Platelet*_v1_*, rate of change in platelet; SCr, serum creatinine; IMV, invasive mechanical ventilation; SCr*_v1_*, rate of change in SCr; WBC*_v1_*, change rate of WBC; CCI Charlson comorbidity index; Total bilirubin*_v1_*, rate of change in total bilirubin; PCT, procalcitonin; Hemoglobin*_v1_*, rate of change in hemoglobin; ALT*_v1_*, change rate of alanine transaminase; ALT*_v-1_*, rate of change in alanine transaminase (pre–blood culture); CRP*_v1_*, rate of change in C-reactive protein; Sodium*_v1_*, change rate of sodium; Platelet*_v-1_*, rate of change in platelet (pre–blood culture).

Continuous variables were presented as medians and interquartile ranges (IQRs), and group comparisons were performed using the Mann–Whitney *U* test. Categorical variables were reported as frequencies and percentages, with group comparisons conducted using the chi-square test or Fisher’s exact test, as appropriate. The eXtreme Gradient Boosting (XGBoost) algorithm was chosen for model development because of its strong predictive performance in sepsis and related disease diagnosis and prognosis (Hou et al., 2020; Wang et al., 2025; Zhou et al., 2024). Hyperparameters were optimized using grid search and cross-validation to ensure model stability. Shapley Additive Explanations (SHAP) were used to interpret and visualize relationships between input variables and outcomes; SHAP values quantified each variable’s contribution to the prediction of 28-day mortality. SHAP-derived variable importance is shown in **Figure 2**. Statistical significance was defined as *P* < 0.05. All statistical analyses and modeling were performed using R (version 4.0.3) and Python (version 3.7.0).

## 3 Results

### 3.1 Baseline characteristics

The AMU and MIMIC-IV cohorts exhibited similar 28-day mortality rates (25.6% vs 25.1%). Across both cohorts, the 28-day mortality group was characterized by older age; higher CCI scores; increased serum creatinine (SCr), prothrombin time (PT), and procalcitonin (PCT) levels; and lower platelet counts (*P* < 0.001). However, analysis of pathogen distribution showed a higher prevalence of Gram-positive bacteria (22.2%, *P* = 0.011) and Gram-negative bacteria (12.3%, *P* = 0.006) in the AMU 28-day mortality group (**Table 1**), whereas the MIMIC-IV 28-day mortality group predominantly exhibited Gram-negative bacteria (4.7%, *P* = 0.003) and fungal infections (3.6%, *P* < 0.001) (**Supplementary Table S3**).

**Table 1:** Baseline characteristics of the AMU dataset.

Variables	Overall (n=1903)	Survival group (n=1416)	Motality group (n=487)	*P* value	SMD
Demography					
Age (years old)	59 (48, 70)	57 (47, 69)	65 (53, 75)	<0.001	0.417
Male (%)	1279 (67.2)	955 (67.4)	324 (66.5)	0.753	0.019
CCI	3 (1, 4)	2 (1, 4)	3 (2, 5)	<0.001	0.473
Laboratory					
WBC (×10^9^/L)	12.1 (8.9, 16.6)	11.9 (9.0, 16.1)	12.7 (8.7, 18.0)	0.078	0.137
Neu (×10^9^/L)	10.1 (7.3, 14.4)	10.0 (7.2, 13.9)	11.0 (7.6, 15.7)	0.019	0.169
Lymp (×10^9^/L)	0.9 (0.6, 1.2)	0.9 (0.6, 1.2)	0.8 (0.5, 1.2)	0.114	0.01
Hemoglobin (g/L)	93 (78, 111)	93 (79, 111)	91 (76, 111)	0.093	0.075
Platelet (×10^9^/L)	150 (100, 215)	158 (107, 222)	127 (71, 188)	<0.001	0.35
ALT (U/L)	44 (26, 97)	43 (26, 93)	47 (26, 106)	0.195	0.222
AST (U/L)	49 (30, 99)	46 (29, 89)	59 (35, 132)	<0.001	0.261
Total bilirubin (mg/dL)	17.3 (10.8, 28.5)	17.0 (10.7, 27.1)	18.2 (11.5, 36.5)	0.012	0.278
SCr (μmol/L)	87 (63, 138)	79 (60, 116)	124 (80, 214)	<0.001	0.406
Sodium (mmol/L)	143 (138, 148)	142 (138, 147)	143 (139, 150)	0.003	0.217
Potassium (mmol/L)	3.9 (3.5, 4.2)	3.9 (3.5, 4.2)	3.8 (3.5, 4.2)	0.792	0.049
Calcium (mmol/L)	2.1 (2.0, 2.2)	2.1 (2.0, 2.2)	2.0 (1.9, 2.2)	<0.001	0.167
PT (s)	13.0 (12.0, 14.6)	12.7 (11.8, 14.0)	13.8 (12.5, 16.2)	<0.001	0.444
INR	1.1 (1.0, 1.3)	1.1 (1.0, 1.2)	1.2 (1.1, 1.4)	<0.001	0.440
CRP (mg/L)	129 (70, 208)	127 (68, 206)	138 (75, 228)	0.036	0.146
PCT (ng/mL)	0.7 (0.2, 2.4)	0.5 (0.2, 1.7)	1.5 (0.3, 4.5)	<0.001	0.271
Therapeutics					
Antibiotic (day)	7 (4, 11)	8 (5, 12)	4 (2, 8)	<0.001	0.660
IMV* (day)	5 (2, 9)	5 (3, 9)	4 (2, 8)	0.001	0.077
RRT* (day)	5 (2, 11)	6 (2, 12)	5 (2, 9)	<0.001	0.081
LOS (day)	7 (3, 15)	7 (3, 15)	6 (3, 13)	0.011	0.083
Pathogen					
Gram^+^ (%)	347 (18.2)	239 (16.9)	108 (22.2)	0.011	0.134
Gram^-^ (%)	174 (9.1)	114 (8.1)	60 (12.3)	0.006	0.142
Fungi (%)	38 (2.0)	26 (1.8)	12 (2.5)	0.505	0.043

CCI, Charlson comorbidity index; WBC, white blood cell; Neu, neutrophil count; Lymp, lymphocyte count; ALT, alanine transaminase; AST, aspartate transaminase; SCr, serum creatinine; PT, prothrombin time; INR, international normalized ratio; CRP, C-reactive protein; PCT, procalcitonin; IMV, invasive mechanical ventilation; RRT, renal replacement therapy; LOS, length of ICU stay; ICU, intensive care unit; Gram+, Gram-positive bacteria; Gram-, Gram-negative bacteria. *Due to substantial data bias, only positive samples were included.

### 3.2 Model development and simplification

**Figure 3:**
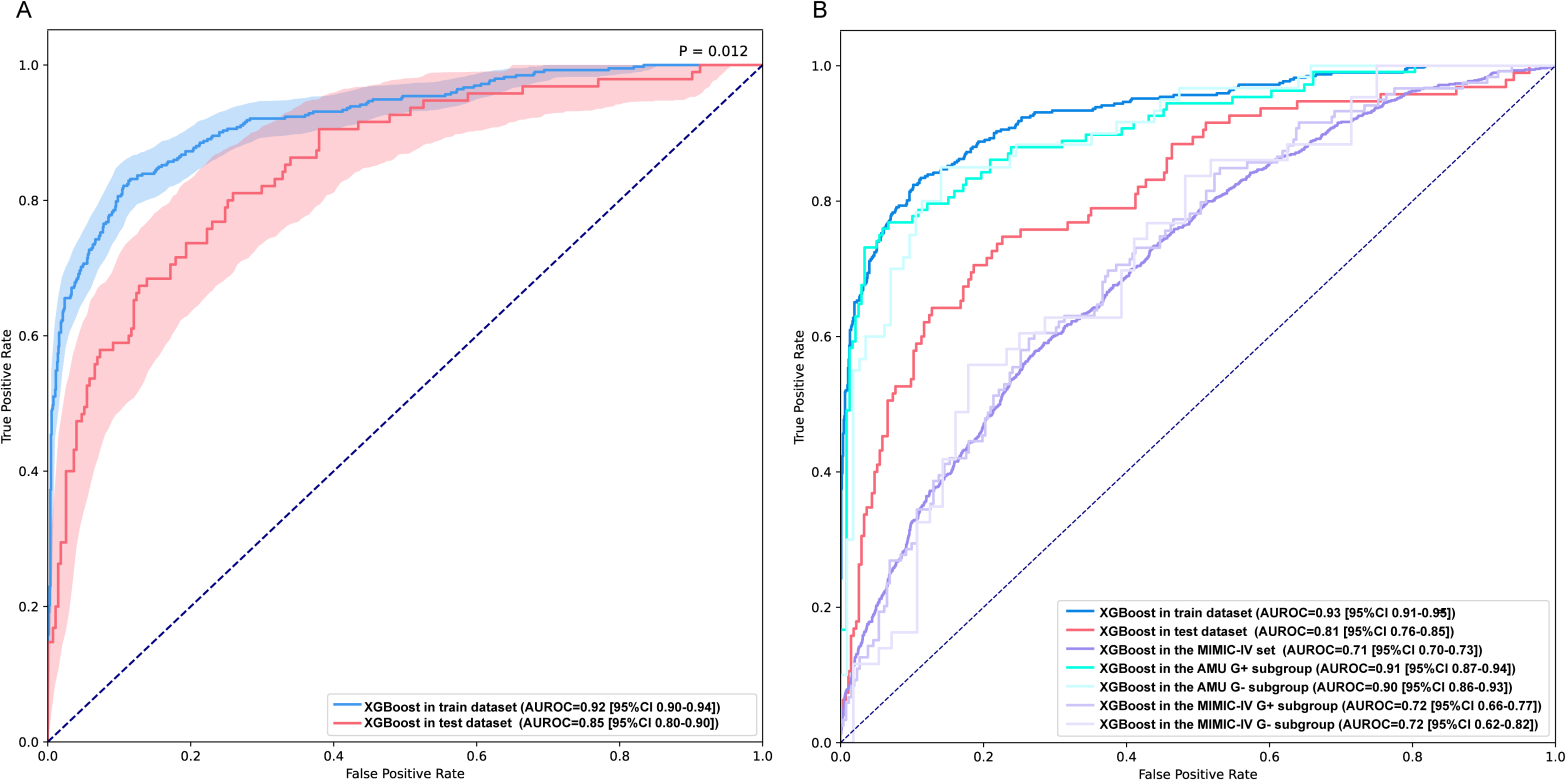
AUROC values of the full and simplified models in the training, test, and MIMIC-IV datasets. **(A)** Comparison of XGBoost model AUROC performance on internal training and test sets. **(B)** Performance of simplified XGBoost model on AMU, MIMIC-IV, and laboratory-confirmed BSI subgroups. test set: internal validation; XGBoost, eXtreme Gradient Boosting; AUROC, Area Under the Receiver Operating Characteristic Curve; AMU, Anhui Medical University; MIMIC-IV, Medical Information Mart for Intensive Care IV; Gram+, Gram-positive bacteria; Gram-, Gram-negative bacteria.

A total of 33 variables were included for model construction. To mitigate overfitting and underfitting and optimize performance, a grid search combined with cross-validation was used to determine the final set of hyperparameters (**Supplementary Table S4**). In the internal training and validation sets, the XGBoost model demonstrated strong discrimination, with AUROC values of 0.92 (95% CI 0.90–0.94) and 0.85 (95% CI 0.80–0.90), respectively (*P* = 0.012; **Figure 3A**). Further analysis showed that AUROC and accuracy increased as variables were sequentially incorporated according to their importance, but both plateaued after 10 variables, indicating that additional variables did not materially improve predictive performance (**Supplementary Figure S3**). Therefore, a simplified 10-variable model was retrained to enhance clinical applicability.

As shown in **Figure 3B** and **Supplementary Figure S4**, the simplified XGBoost consistently outperformed the other approaches, achieving AUROCs of 0.93 (95% CI 0.91–0.95) in the training set, 0.81 (95% CI 0.76–0.85) in the internal validation set, and 0.71 (95% CI 0.70–0.73) in the MIMIC-IV external validation set.

In the Gram-positive and Gram-negative subgroups, the simplified model achieved AUROCs of 0.91 (95% CI 0.87–0.94) and 0.90 (95% CI 0.86–0.93) in the AMU dataset, and 0.72 (95% CI 0.66–0.77) and 0.72 (95% CI 0.62–0.82) in the MIMIC-IV dataset, respectively. These values were all higher than those observed in the overall cohort.

To enable fair comparisons, we computed AUROC, sensitivity, specificity, positive predictive value (PPV), and negative predictive value (NPV) for all models at the optimal cutoff. Across datasets, the simplified XGBoost model consistently outperformed the comparator models—LR, k-nearest neighbors (KNN), random forest (RF), support vector machine (SVM), and artificial neural network (ANN) in terms of AUROC, sensitivity, specificity, and NPV (**Supplementary Table S5**).

Decision curve analysis (DCA) showed that the simplified XGBoost model yielded stable and relatively high net benefit on internal data, particularly in the training set; on the external dataset, net benefit was lower and remained positive, and therefore clinically useful, only at low threshold probabilities (**Supplementary Figure S5A**). Overall calibration results showed that, in the training set, the simplified XGBoost model’s calibration curve was closest to the ideal line and performed best; in the internal validation set it deviated only slightly, indicating acceptable calibration. In the external validation set (MIMIC-IV), the curve lay largely below the ideal line, suggesting overall risk overestimation, although the curve was smooth and the magnitude of deviation was relatively small (**Supplementary Figure S5B**). For the comparator models (LR, KNN, RF, SVM, and ANN), adherence to the ideal line was acceptable in the low-to-intermediate predicted-probability range but worsened at higher probabilities, and systematic overestimation with reduced stability was observed in the external validation set (**Supplementary Figures S6A–E**). Overall, the simplified XGBoost model showed better probability calibration than the other models in the training and internal validation sets; on external data it still tended to overestimate risk, but the bias was smaller.

### 3.3 Model interpretation

**Figure 4:**
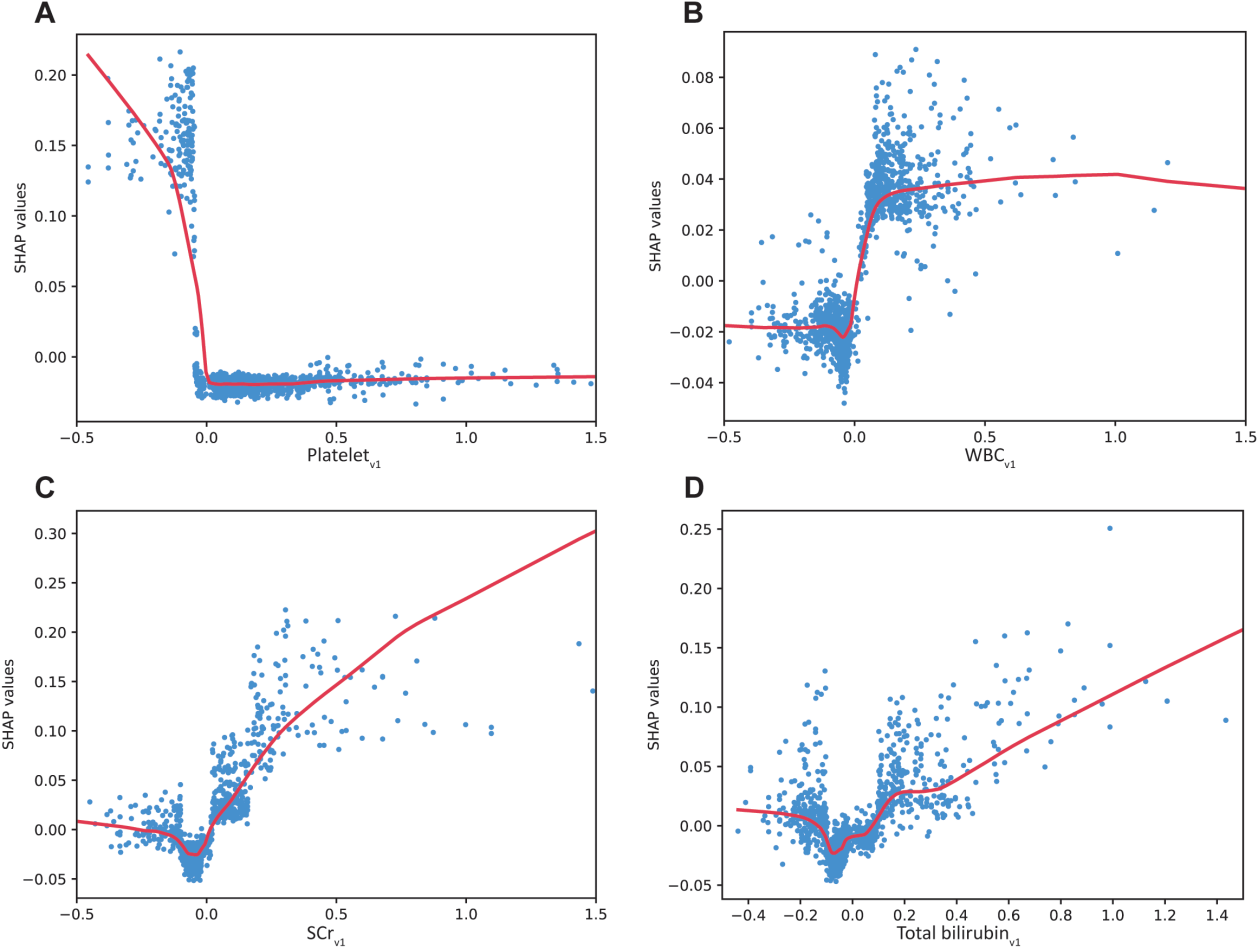
Partial dependence plots of variables on 28-day mortality. Partial dependence plots (PDPs) illustrate variable effects on 28-day mortality. **(A)** Effect of platelet*_v1_* on 28-day mortality. **(B)** Association between WBC*_v1_* and 28-day mortality. **(C)** Comparison of SCr*_v1_* and 28-day mortality. **(D)** Influence of total bilirubin*_v1_* on 28-day mortality. Platelet*_v1_*, rate of change in platelet; WBC*_v1_*, rate of change in WBC; SCr*_v1_*, rate of change in SCr; Total bilirubin*_v1_*, rate of change in total bilirubin.

To further investigate the impact of individual variable changes on 28-day mortality in patients with ICU-BSIs, partial dependence plots (PDPs) were generated for the 10 most influential continuous variables (**Figure 4**; **Supplementary Figure S7**). SHAP values were used to quantify each variable’s effect on the predicted probability of 28-day mortality, with positive values indicating increased risk and negative values indicating a protective effect. The top 10 variables, ranked in descending order of importance, were: duration of antibiotic use; average rate of change in platelet count; SCr; duration of invasive mechanical ventilation (IMV); average rate of change in SCr, average rate of change in white blood cell count (WBC), CCI; total bilirubin; PCT; and average rate of change in hemoglobin. **Figures 4A–D** shows that dynamic rates of change in laboratory parameters were closely associated with 28-day mortality in patients with ICU-BSIs. A decreasing platelet trajectory was associated with a marked increase in mortality risk, whereas stable or rising trends were linked to lower risk (A). WBC dynamics followed a nonlinear pattern: decreases were associated with relatively lower risk, but excessive increases were accompanied by higher risk (B). Changes in serum creatinine demonstrated an approximately linear positive association with mortality, indicating that worsening renal function was strongly related to poor prognosis (C). Similarly, increases in total bilirubin were associated with a gradual rise in mortality risk, whereas stable or lower levels suggested a more favorable prognosis (D).

As shown in **Supplementary Figure S7**, the SHAP dependence analysis revealed interpretable threshold effects of several key clinical variables on 28-day mortality among patients with ICU-BSIs. (1) A. Antibiotic duration: Treatment lasting more than two days was associated with a significant reduction in mortality risk, suggesting a protective effect of adequate therapy. (2) B. Scr: > 110 μmol/L was linked to a marked increase in mortality risk, reflecting the adverse prognostic impact of renal dysfunction. (3) C. IMV: Mortality risk progressively increased once IMV > 24 hours, indicating that longer respiratory support was associated with worse outcomes. (4) D. CCI: > 3 were associated with a substantial rise in mortality risk, consistent with the influence of comorbidity burden on adverse outcomes. (5) E. PCT: > 0.1 ng/mL were associated with increased mortality, consistent with its established role as a biomarker of infection severity. (6) F. Hemoglobin: Stable levels corresponded to the lowest risk, whereas marked fluctuations—either increases or decreases—were associated with elevated mortality risk.

Furthermore, this study explored interactions among common clinical variables, revealing the following relationships with 28-day mortality. **Supplementary Figure S8** presents SHAP-based interaction dependence plots (y-axis: SHAP value; higher values indicate a greater positive contribution to the risk of 28-day mortality). The findings were as follows: (1) S8A: A decreasing platelet change rate was associated with higher risk, and this effect was intensified when the WBC change rate further decreased; increases in the platelet change rate alone had a limited risk-reducing effect. (2) S8B: An increasing WBC change rate was associated with greater risk, with a stronger effect at higher PCT levels; risk rose sharply when WBC decreased while PCT increased, indicating a synergistically adverse interaction. (3) S8C: Fluctuations of SCr within the normal range had little impact; once SCr exceeded the normal threshold, even slightly, risk increased rapidly, and when accompanied by a positive SCr change rate the risk further escalated, indicating a threshold effect and interaction. (4) S8D: Longer duration of IMV was associated with higher risk, whereas a longer course of antibiotics was associated with lower risk, especially when IMV duration was prolonged, suggesting a mitigating effect. Taken together, these plots indicate nonlinear and interactive effects of key variables on 28-day mortality: decreased platelets combined with unfavorable WBC changes, high PCT concurrent with unfavorable WBC changes, SCr above threshold with continued increase, and prolonged IMV with insufficient antibiotic duration were each associated with higher risk, whereas adequately sustained antibiotic therapy partially offset the risk associated with prolonged IMV in some scenarios.

To evaluate the performance of individual variables and their rate-of-change transformations, **Supplementary Figure S9** compares the AUROC of single variables in the two cohorts. The x-axis shows 1–specificity (FPR) and the y-axis sensitivity (TPR); the diagonal dashed line denotes the no-discrimination reference, and the colored bands indicate 95% confidence intervals. In AMU (panel A), most curves lie above the diagonal, indicating moderate discrimination, with single-variable AUROCs of approximately 0.55–0.72; rate-of-change features generally outperform their corresponding baseline-level variables. In MIMIC-IV (panel B), a similar pattern is observed, although AUROCs on the external cohort cluster around 0.57–0.64, indicating weaker discrimination. The relative ranking of variables is broadly consistent with AMU, suggesting external consistency and robustness.

### 3.4 Web–based tool

Although the model demonstrates relatively high interpretability, its clinical applicability remains constrained by the inherent computational complexity of the machine learning framework adopted in this study. To address this limitation, we developed a web–based prognostic prediction system for ICU-BSIs, which has been made openly available on GitHub (https://github.com/jaser1314/simulation-software-for-zhou) to facilitate further exploration and validation by researchers. The program is designed to be executed locally, thereby avoiding the use or transmission of any patient-level data, and it supports cross-platform deployment (iOS, Windows, and Android), enhancing its usability across diverse computational environments.

## 4 Discussion

This study successfully developed and validated a machine learning–based prognostic model for ICU-BSIs, demonstrating strong predictive performance across diverse clinical settings. In contrast to single-center models (Liu et al., 2023; Zhang et al., 2022), our XGBoost model, trained on data from AMU (China) and MIMIC-IV (USA), is a valuable tool for predicting 28-day mortality in high-risk patients, with the goal of improving care and outcomes.

Existing prognostic models for critical patients typically depend on static data such as demographic characteristics, pathogen profiles, laboratory values at a single time point, and clinical treatments (Rahman et al., 2024; Schwab et al., 2018; Taneja et al., 2021; Zhang et al., 2024, 2022). We addressed a critical gap in the literature by incorporating dynamic trends in clinical laboratory indicators, particularly during the blood culture collection period, thus significantly improving predictive performance compared to models relying on fixed reference ranges. The model is intended for risk assessment at the time of clinical suspicion, with the prediction time defined as the blood culture timestamp (*t*_0_). In real-time clinical use, all dynamic features are computed relative to the fixed baseline at *t*_0_, and the risk estimate can be prospectively updated as subsequent observations become available. The incorporation of rate-of-change transformations of laboratory values was demonstrated to enhance the model’s capacity to capture the temporal dynamics of disease progression, thereby leading to a significant improvement in overall predictive performance. In this study, the newly constructed variable achieved a higher AUROC than the original variable, demonstrating superior predictive value. These findings suggest that integrating interpretability methods with variable reconstruction may help bridge the gap between “black-box” models and clinical practice, thereby providing methodological support and theoretical rationale for potential risk stratification and early intervention.

Numerous variables associated with mortality at various time points have been investigated in previous research (Chen et al., 2022; Ye et al., 2024). Aligning with established clinical knowledge, our analysis identified duration of antibiotic use, platelet count, SCr level, duration of invasive mechanical ventilation, and CCI score as the top variables associated with 28-day mortality in both datasets, highlighting the complex interaction of factors affecting patient outcomes in ICU-BSIs. Importantly, the impact of antibiotic duration on 28-day mortality appears to be time-dependent, with patients potentially benefiting only after a certain period of administration. This suggests that early-stage mortality may be less responsive to antibiotics alone, likely due to the delayed onset of their efficacy. It follows that the early mortality risk is likely more associated with the patient’s baseline health and immune function rather than being solely determined by the therapeutic effects of antibiotics. Additionally, the significance of platelet count (reflecting immune function) and SCr (reflecting kidney health) highlights the importance of these host factors in determining patient outcomes. Previous studies were replicated with lower platelet counts increasing mortality (Ye et al., 2024); however, our results indicated that increased platelets showed no effect. One potential cause could be that the apoptosis of platelets is directly linked to increased bloodstream infection (BSI) and poor patient outcomes (Gründler et al., 2014; Kraemer et al., 2012). Multiple studies have demonstrated that baseline comorbidities exert a significant influence on the prognosis of patients with infection (Ibarz et al., 2024). Moreover, both excessive immune activation and immune suppression have been linked to adverse outcomes, underscoring the critical role of immune homeostasis in determining disease trajectory (Annane et al., 2005; Singer et al., 2016). In addition, sepsis-associated acute kidney injury (AKI) and hepatic dysfunction have been consistently shown to correlate with increased mortality (Liu et al., 2020; Tanaka et al., 2022; White et al., 2023), highlighting the central contribution of progressive multi-organ dysfunction to poor outcomes. Notably, dynamic changes in laboratory parameters—including creatinine, bilirubin, platelets, and WBC counts—not only constitute essential components of established severity scoring systems but also serve as important predictors of clinical prognosis (Varga et al., 2025; Wang et al., 2024).

The number of variables incorporated into the high-frequency modeling framework ranged from 11 to 30, encompassing key physiological and clinical indicators such as age, lactate levels, systolic blood pressure, heart rate, body temperature, oxygen saturation (SpO2), Glasgow Coma Scale (GCS) score, ventilator status, creatinine levels, and platelet count (Hou et al., 2020; Zhang et al., 2022, 2023). While the initial XGBoost model included 33 clinical variables, the simplified model, incorporating only the top 10 variables identified through SHAP analysis, maintained robust accuracy. This simplification process is crucial for enhancing the model’s practicality and facilitating its implementation in clinical settings. Furthermore, the simplified XGBoost model exhibited high performance, with an AUROC of 0.93 in the training set, 0.81 in the internal validation set, and 0.71 in the MIMIC-IV validation set, indicating improved results for bacterial subgroup identification. As a retrospective, exploratory study, we acknowledge that including variables available only after the prediction time (e.g., post-blood culture maxima) may introduce look-ahead bias and inflate internal AUROC; accordingly, we treated these as exploratory descriptors of the full-stay risk signal rather than for real-time inference, and we will anchor prediction at the blood culture timestamp (*t_0_*) in prospective multicenter validation, restrict features to data available on or before *t_0_*, perform recalibration, and quantify real-world lead time and alert burden to optimize thresholding and alert management.

The current study presents several potential implications for clinical practice and future research. First, the developed model may facilitate the early identification of patients at elevated risk of adverse outcomes, enabling the implementation of timely and targeted interventions to potentially improve patient prognosis. Second, the model offers a framework for personalized, dynamic monitoring of disease progression, allowing clinicians to improve treatment strategies based on individual risk profiles and optimize therapeutic efficacy. Third, the identification and ranking of key prognostic variables provide valuable insights into the complex pathophysiology of ICU-BSIs, which are crucial for the rational design and evaluation of novel clinical interventions.

In conclusion, we developed a simple, scalable, and user-friendly prognostic model for ICU-BSIs that was rigorously validated and demonstrated robust predictive accuracy across diverse clinical settings. By combining machine learning with readily available clinical data, this model provides a valuable and interpretable tool for predicting outcomes in patients with ICU-BSIs. Our findings underscore the importance of dynamic monitoring and individualized assessment in managing this high-risk population, thereby facilitating improved clinical decision-making and enhancing patient outcomes.

## 5 Limitations

This study has several limitations. First, the external validation in the MIMIC-IV dataset showed a slight decline in performance compared with internal validation, which may be explained by differences in data collection practices, patient populations, missing key variables (e.g., PCT and bilirubin), domain shift due to cross-center heterogeneity, and variations in pathogen distribution and antimicrobial resistance patterns. Second, although the validation in MIMIC-IV, which included multiple pathogen subgroups, demonstrated good applicability to other causative microorganisms, further external validation in diverse datasets is required. Subgroup-specific validation of cutoffs was not performed due to limited sample sizes after stratification and predictor unavailability in the external cohort; future work will prioritize multicenter recalibration across age, sex, and comorbidity strata. We did not perform prospective, multicenter external validation and recalibration, which precludes quantifying the model’s real-world lead time and alert burden. Because our results are highly specific to patients with ICU-BSIs, the model’s generalizability to other ICU populations or to general hospitalized patients with bloodstream infection (BSI) remains limited. Finally, our model was developed based on retrospective data through simulated dynamic analyses, and the simplified extraction of dynamic variable information in this study may still pose certain limitations. Future research may focus on refining the extraction formulas to enhance their scientific rigor and improve computational efficiency, and prospective validation will be required to substantiate the robustness and validity of this approach.

## Data Availability

The original contributions presented in the study are included in the article/**Supplementary Material**. Further inquiries can be directed to the corresponding authors.
