# Supplementary material for: Personalized machine learning–based prognostic model for ICU-acquired bloodstream infections
Source: Front Cell Infect Microbiol. 2025 Oct 29;15:1636886. doi: 10.3389/fcimb.2025.1636886 (PMC12605174; doi:10.3389/fcimb.2025.1636886)
Supplement: Supplementary file 1 [file SupplementaryFile1.docx]

## Table S1. Variable data types, directions and abbreviations.

| Variables | Data types | Extract directions | Extract durations | Abbreviations | |
| --- | --- | --- | --- | --- | --- |
| **Demographic** |  |  |  |  | |
| Age | Numerical | - | - | | Age |
| Male | Binary | - | - | | Male |
| **Comprehensive score** |  |  |  | |  |
| Charlson Comorbidity Index | Numerical | - | - | | CCI |
| **Laboratory** |  |  |  | |  |
| White blood cell count | Numerical | Max | Within 24 hours | | WBC |
| Neutrophil count | Numerical | Max | Within 24 hours | | Neu |
| Lymphocyte count | Numerical | Min | Within 24 hours | | Lymp |
| Hemoglobin | Numerical | Min | Within 24 hours | | - |
| Platelet | Numerical | Min | Within 24 hours | | - |
| C-reactive protein | Numerical | Max | Within 24 hours | | CRP |
| Procalcitonin | Numerical | Max | Within 24 hours | | PCT |
| Serum Creatinine | Numerical | Max | Within 24 hours | | SCr |
| Sodium | Numerical | Min | Within 24 hours | | - |
| Potassium | Numerical | Min | Within 24 hours | | - |
| Calcium | Numerical | Min | Within 24 hours | | - |
| Alanine transaminase | Numerical | Max | Within 24 hours | | ALT |
| Aspartate transaminase | Numerical | Max | Within 24 hours | | AST |
| Total bilirubin | Numerical | Max | Within 24 hours | | - |
| Prothrombin time | Numerical | Max | Within 24 hours | | PT |
| International standardized ratio | Numerical | Max | Within 24 hours | | INR |
| **Therapeutic indicators and prognosis** |  |  |  | |  |
| Renal replacement therapy | Binary | - | Daily | | RRT |
| Invasive mechanical ventilation | Binary | - | Daily | | IMV |
| Length of ICU stay | Numerical | - | - | | LOS |
| 28-day mortality (%) | Binary | - | - | | - |

We addressed missing data using the Multivariate Imputation by Chained Equations (MICE) framework, generating 50 imputed datasets with predictive mean matching for continuous variables, logistic regression for binary variables, and polytomous regression for categorical variables, with a fixed seed (123) to ensure reproducibility. This approach minimized bias, preserved statistical power, and ensured stable model development.

## Table S2. Sample size considerations for binary outcome models.

|  | Samp size | Shrinkage | Parameter | CS Rsq | Max Rsq | Nag Rsq | EPP |
| --- | --- | --- | --- | --- | --- | --- | --- |
| Criteria 1 | 570 | 0.900 | 10 | 0.1449 | 0.676 | 0.214 | 14.31 |
| Criteria 2 | 269 | 0.811 | 10 | 0.1449 | 0.676 | 0.214 | 6.75 |
| Criteria 3 | 289 | 0.900 | 10 | 0.1449 | 0.676 | 0.214 | 7.25 |
| Final | 570 | 0.900 | 10 | 0.1449 | 0.676 | 0.214 | 14.31 |

Abbreviations: CS Rsq, Cox–Snell R²; Max Rsq, Maximum R²; Nag Rsq, Nagelkerke R²; EPP, Events per predictor.

## Table S3. Baseline data of patients in the MIMIC-IV data set.

| Variables | Overall | Survival group | Motality group | *P* value | SMD |
| --- | --- | --- | --- | --- | --- |
|  | (n=3496) | (n=2617) | (n=879) |  |  |
| **Demography** |  |  |  |  |  |
| Age (years old) | 67 (55, 77) | 64 (53, 75) | 73 (61, 83) | <0.001 | 0.492 |
| Male (%) | 2082 (59.6) | 1567 (59.9) | 515 (58.6) | 0.526 | 0.026 |
| CCI | 5 (3, 7) | 4 (2, 7) | 6 (4, 8) | <0.001 | 0.583 |
| **Laboratory** |  |  |  |  |  |
| WBC (×10^9^/L) | 13.0 (9.5, 17.5) | 12.6 (9.3, 17.0) | 14.1(10.2, 19.3) | <0.001 | 0.223 |
| Hemoglobin (g/L) | 93 (81, 108) | 94 (82, 110) | 89 (78, 104) | <0.001 | 0.227 |
| Platelet (×10^9^/L) | 166 (110, 230) | 172 (119, 236) | 149 (87, 213) | <0.001 | 0.232 |
| SCr (μmol/L) | 88 (62, 159) | 80 (62, 133) | 133 (80, 239) | <0.001 | 0.321 |
| Sodium (mmol/L) | 138 (135, 142) | 138 (135, 142) | 138 (134,143) | 0.431 | 0.064 |
| Potassium (mmol/L) | 3.7 (3.4, 4.0) | 3.7 (3.4, 4.0) | 3.8 (3.4, 4.1) | <0.001 | 0.188 |
| Calcium (mmol/L) | 2.1 (1.9, 2.2) | 2.1 (1.9, 2.2) | 2.0 (1.9, 2.2) | 0.169 | 0.058 |
| PT (s) | 14.2 (12.8, 17.0) | 13.9 (12.6, 16.1) | 15.5(13.4, 21.0) | <0.001 | 0.400 |
| INR | 1.3 (1.2, 1.6) | 1.3 (1.1, 1.5) | 1.4 (1.2, 1.9) | <0.001 | 0.393 |
| **Therapeutics** |  |  |  |  |  |
| Antibiotic (day) | 4 (2, 6) | 4 (2, 7) | 3 (2, 5) | <0.001 | 0.232 |
| IMV (day) | 1 (0, 4) | 1 (0, 4) | 2 (0, 5) | <0.001 | 0.093 |
| RRT* (day) | 5 (2, 10) | 6 (3, 11) | 4 (2, 8) | <0.001 | 0.213 |
| LOS (day) | 5 (2, 11) | 6 (2, 12) | 5 (2, 9) | <0.001 | 0.295 |
| **Pathogen** |  |  |  |  |  |
| Gram^+^ (%) | 387 (11.1) | 283 (10.8) | 104 (11.8) | 0.441 | 0.032 |
| Gram^-^ (%) | 108 (3.1) | 67 (2.6) | 41 (4.7) | 0.003 | 0.113 |
| Fungi (%) | 58 (1.7) | 26 (1.0) | 32 (3.6) | <0.001 | 0.177 |

CCI, charlson comorbidity index; WBC, white blood cell count; SCr, serum creatinine; PT, prothrombin time; INR, international normalized ratio; IMV, invasive mechanical ventilation; RRT, renal replacement therapy; LOS, length of ICU stay; ICU, intensive care unit; Gram^+^, Gram-positive bacteria; Gram^-^, Gram-negative bacteria; *, Due to substantial data bias, only positive samples were included.

## Table S4. Hyperparameter search ranges and final values of each model.

| Models | Hyper-parameters | Search Range | Final value |
| --- | --- | --- | --- |
| K-nearest neighbor | n_neighbors | 2-11 | 2 |
|  | p | 1-5 | 1 |
| Random Forest | n_estimators | 2-5 | 5 |
|  | max_features | 1-5 | 3 |
|  | min_samples_split | 2-5 | 5 |
| Support vector machine | C | 0-1 | 1 |
|  | gamma | 0.0001-0.05 | 0.01 |
| Artificial neural network | alpha | 0.00001-0.001 | 0.001 |
|  | max_iter | 10-5000 | 500 |
| XGBoost | max_depth | 5-11 | 7 |
|  | n_estimators | 30-90 | 90 |
|  | learning_rate | 0.001-0.1 | 0.01 |
|  | gamma | 0.001-0.1 | 0.1 |
|  | min_child_weight | 5-11 | 11 |

XGBoost, eXtreme Gradient Boosting; MIMIC-IV, Medical Information Mart for Intensive Care IV.

## Table S5. Predictive performance of simple models with optimal truncation thresholds.

| Method | Data set | Cut-off value | | AUROC | Sensitivity | Specificity | PPV | NPV |
| --- | --- | --- | --- | --- | --- | --- | --- | --- |
| XGBoost | Train | 0.27 | 0.92 (0.90-0.94) | | 0.84 | 0.85 | 0.66 | 0.94 |
| XGBoost | Test | 0.32 | 0.83 (0.76-0.87) | | 0.67 | 0.88 | 0.66 | 0.89 |
| XGBoost | MIMIC-IV | 0.38 | 0.73 (0.71-0.75) | | 0.74 | 0.61 | 0.39 | 0.87 |
| LR | Train | 0.26 | 0.76 (0.73-0.78) | | 0.79 | 0.61 | 0.41 | 0.90 |
| LR | Test | 0.25 | 0.77 (0.72-0.82) | | 0.85 | 0.58 | 0.42 | 0.92 |
| LR | MIMIC-IV | 0.39 | 0.70 (0.68-0.72) | | 0.72 | 0.59 | 0.37 | 0.86 |
| KNN | Train | 0.25 | 0.84 (0.82-0.86) | | 0.89 | 0.62 | 0.44 | 0.94 |
| KNN | Test | 0.38 | 0.74 (0.68-0.80) | | 0.60 | 0.80 | 0.51 | 0.85 |
| KNN | MIMIC-IV | 0.38 | 0.64 (0.62-0.66) | | 0.71 | 0.53 | 0.34 | 0.84 |
| RF | Train | 0.34 | 0.99 (0.99-1.00) | | 0.96 | 0.92 | 0.81 | 0.99 |
| RF | Test | 0.40 | 0.80 (0.76-0.84) | | 0.66 | 0.82 | 0.56 | 0.88 |
| RF | MIMIC-IV | 0.41 | 0.69 (0.67-0.71) | | 0.71 | 0.58 | 0.36 | 0.85 |
| SVM | Train | -0.99 | 0.85 (0.82-0.87) | | 0.82 | 0.79 | 0.57 | 0.93 |
| SVM | Test | -0.88 | 0.74 (0.68-0.80) | | 0.69 | 0.74 | 0.49 | 0.88 |
| SVM | MIMIC-IV | -0.72 | 0.66 (0.64-0.68) | | 0.66 | 0.61 | 0.36 | 0.84 |
| ANN | Train | 0.33 | 0.79 (0.76-0.81) | | 0.74 | 0.71 | 0.47 | 0.89 |
| ANN | Test | 0.30 | 0.80 (0.76-0.85) | | 0.82 | 0.69 | 0.48 | 0.92 |
| ANN | MIMIC-IV | 0.47 | 0.70 (0.68-0.72) | | 0.71 | 0.60 | 0.38 | 0.86 |

AUROC, area under the receiver operating characteristic curve; PPV, positive predictive value; NPV, negative predictive value.


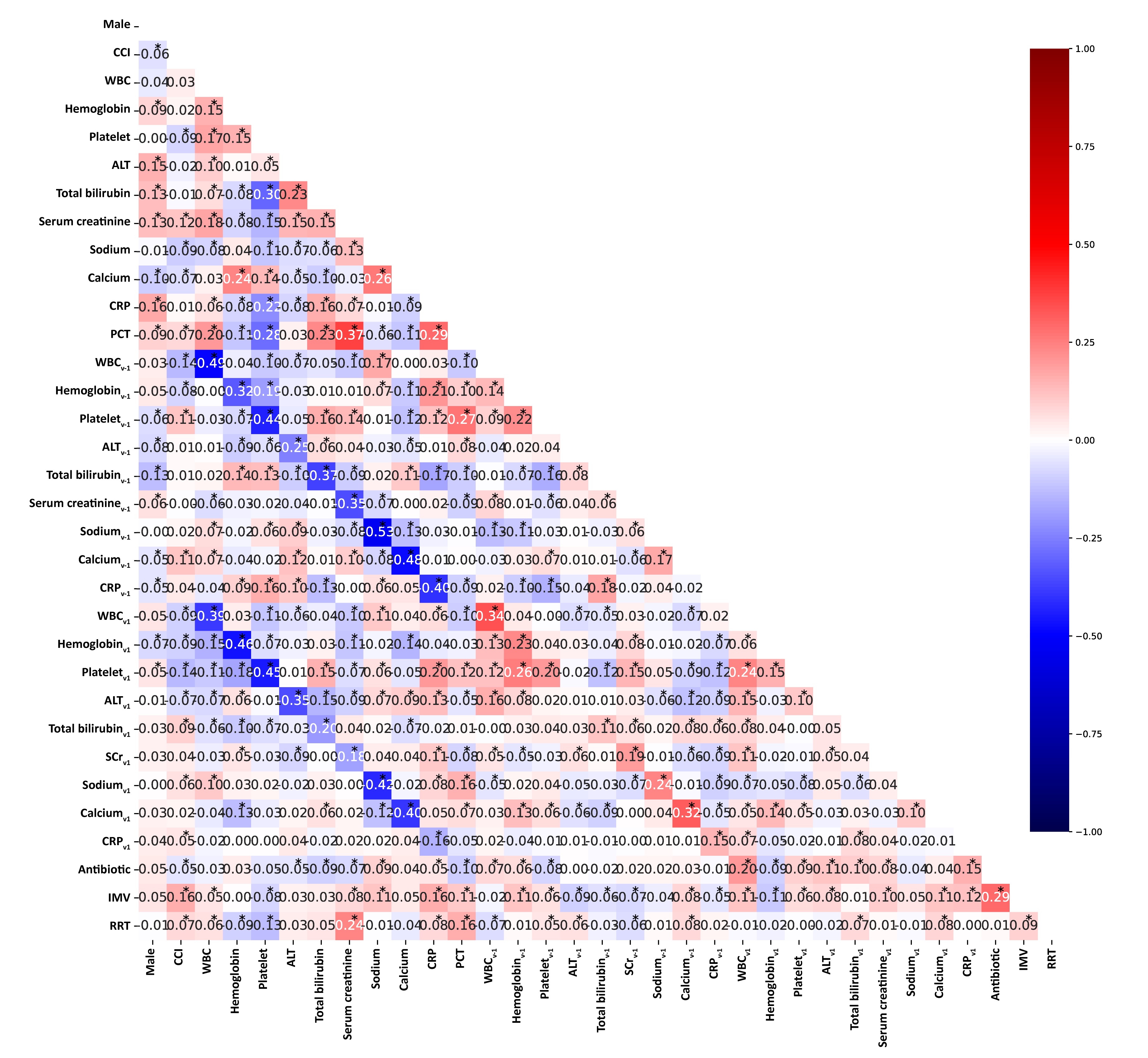


## Figure S1. Spearman correlation heatmap of clinical variables.

The Spearman correlation coefficient (ρ) ranges from -1 to 1, with values closer to 0 indicate a weaker monotonic association between the variables.


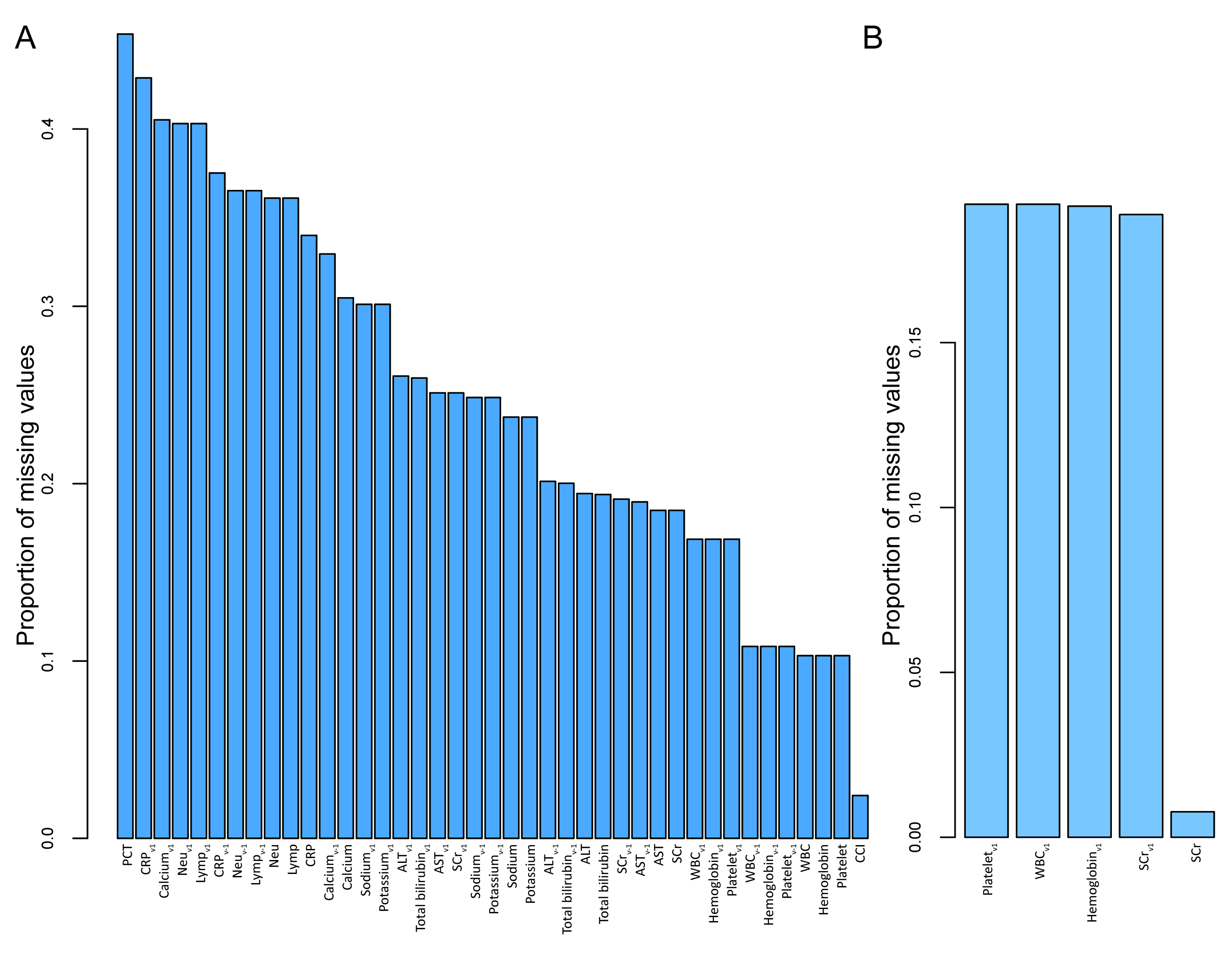


## **Figure S2. Histograms of variable distributions in AMU and MIMIC-IV datasets (≤ 50% missing data).**

A. AMU dataset: Variable distributions (≤ 50% missing data). B. MIMIC-IV dataset: Variable distributions (≤ 50% missing data).


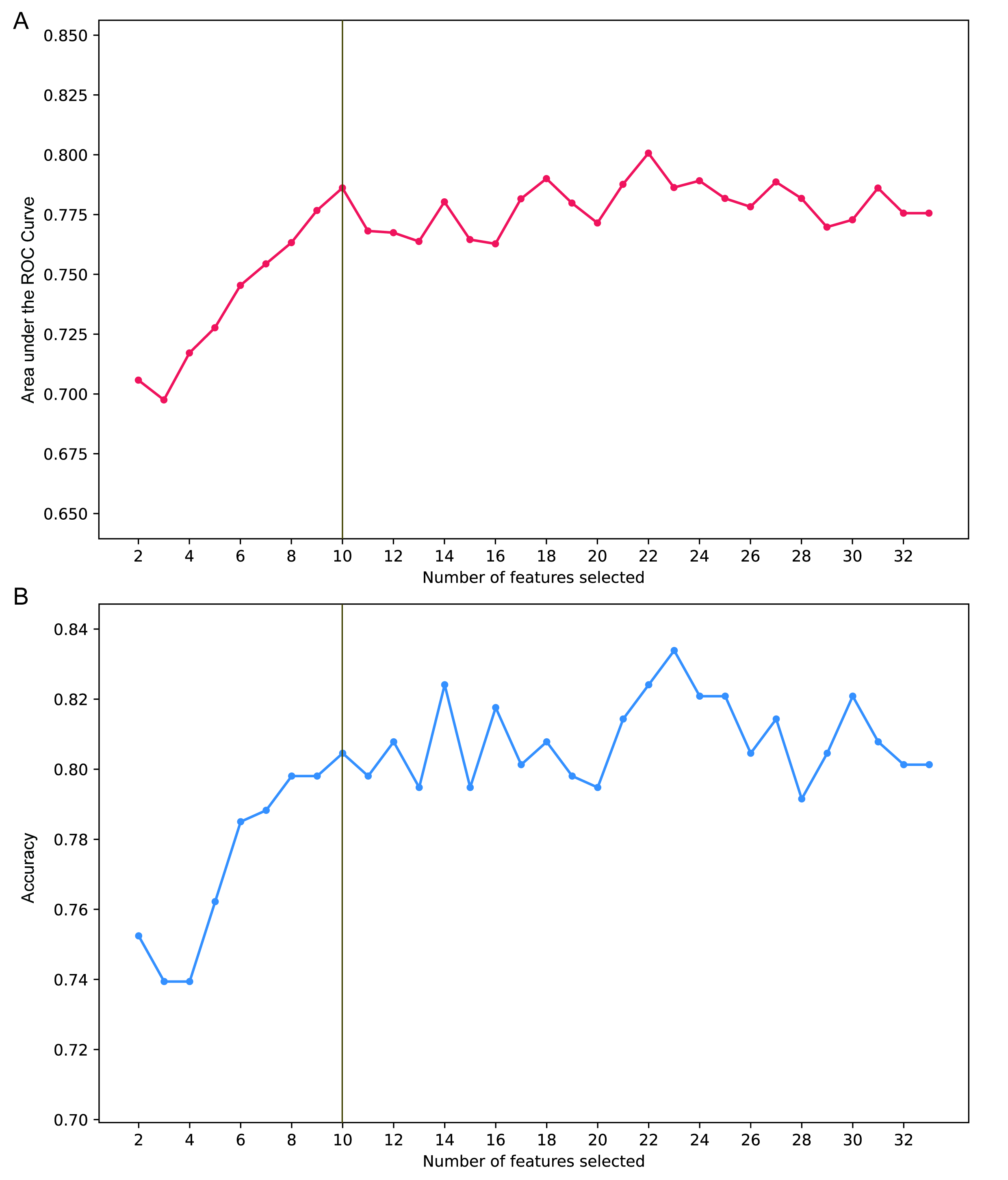


## Figure S3. Dynamic performance of XGBoost model with varying variable number.

A. Dynamic AUROC of XGBoost model with varying variable number. B. Dynamic accuracy of XGBoost model with varying variable number.


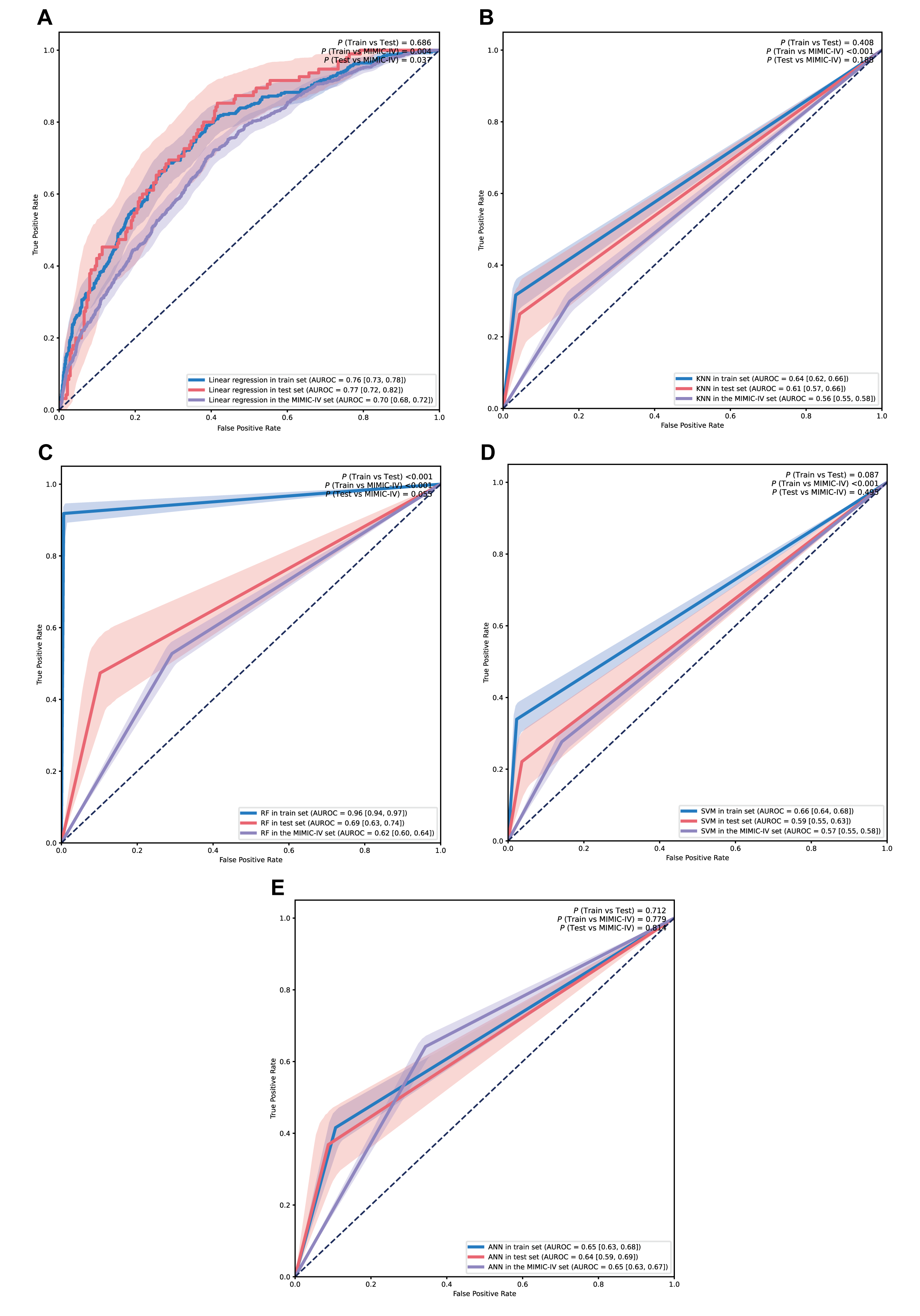


## Figure S4. AUROC of different models accross datasets.

A. Linear regression: AUROC accross datasets. B. KNN: AUROC accross datasets. C. RF: AUROC accross datasets. D. SVM: AUROC accross datasets. E. ANN: AUROC accross datasets.


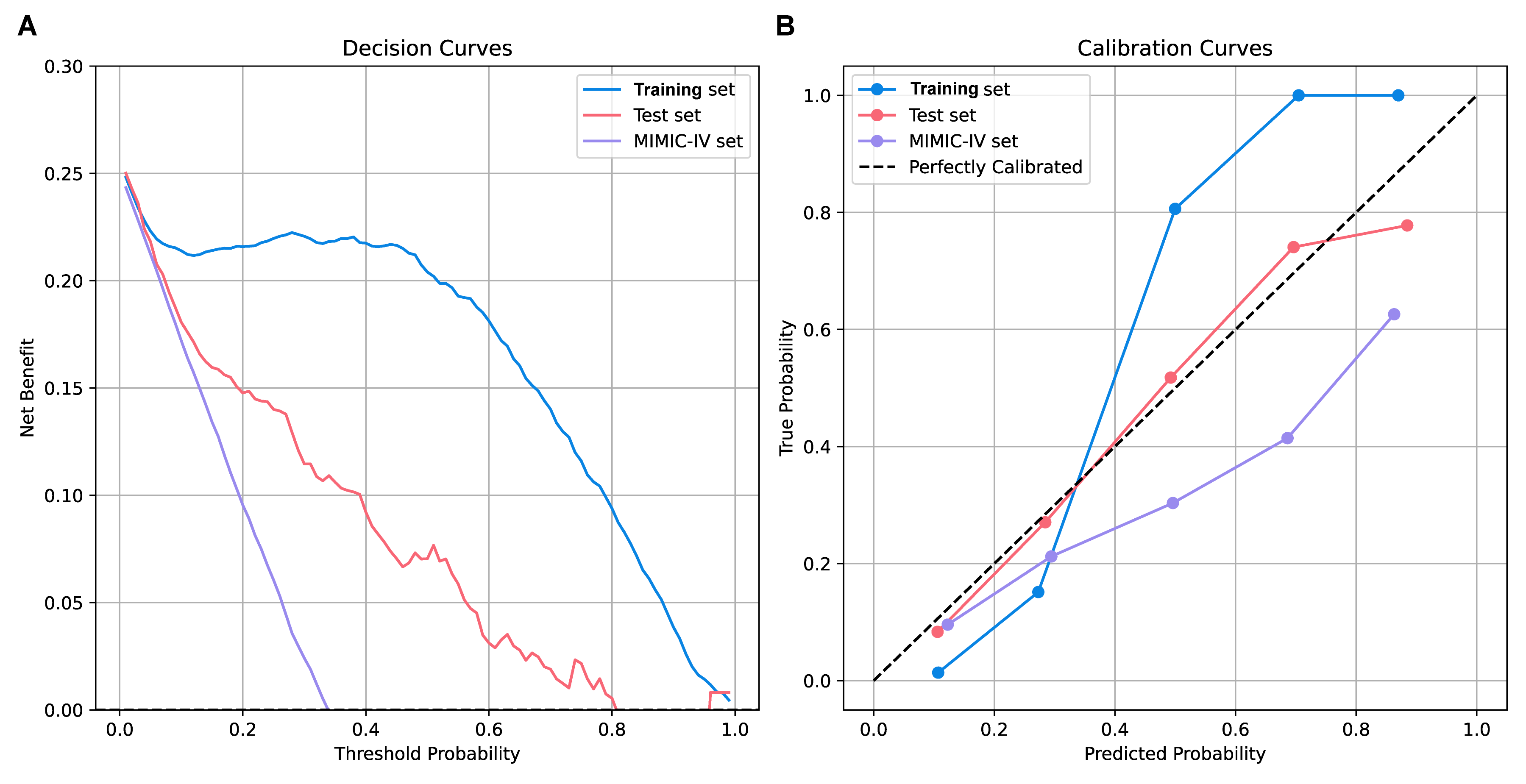


## Figure S5. Decision and calibration curves of simplified XGBoost model accross datasets.

A. Decision curve analysis (DCA) accross datasets. B. Calibration curves accross deatasets.


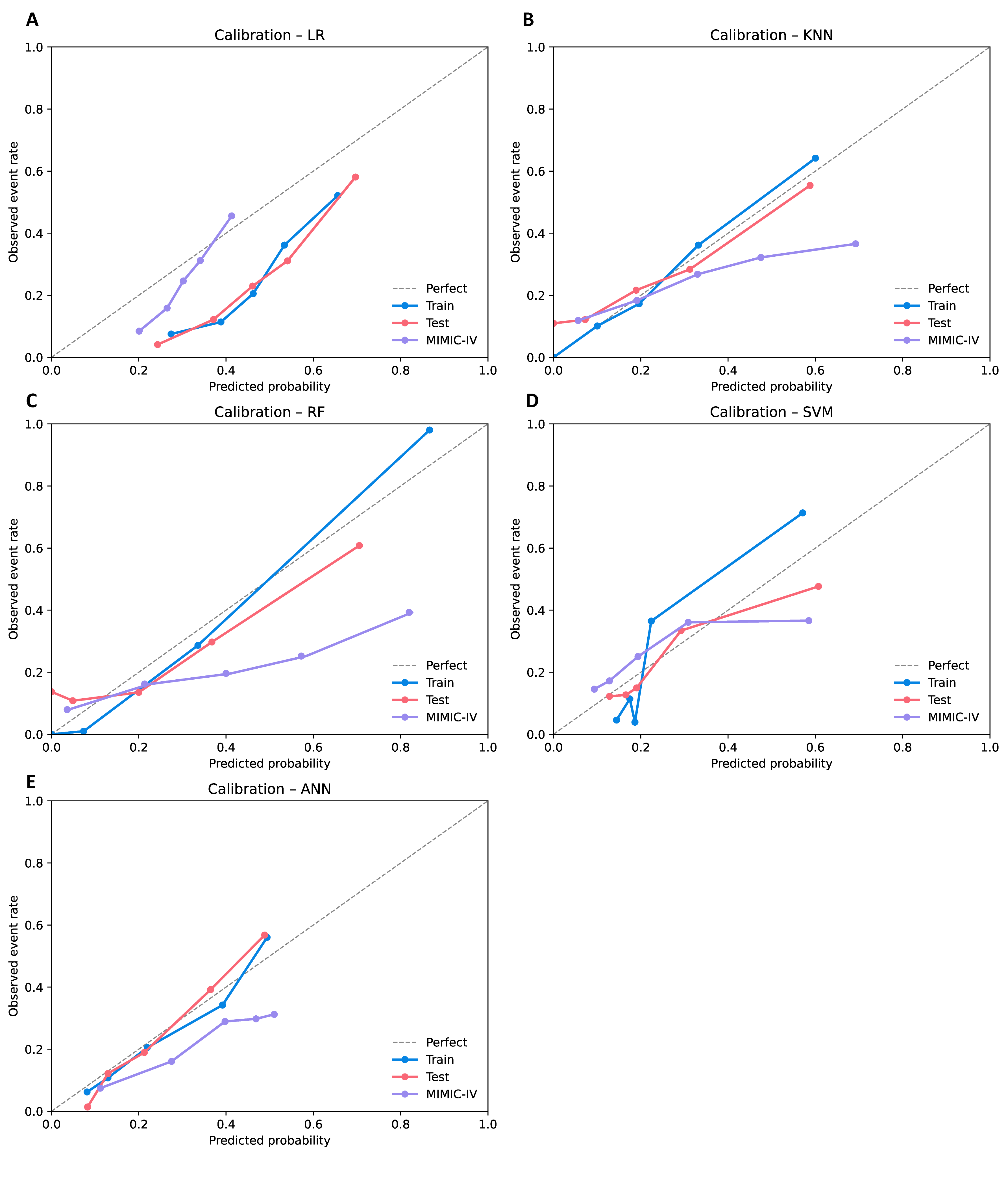


## Figure S6. Calibration curves of LR(A), KNN(B), RF(C), SVM(D) and ANN(E) accross deatasets.

**
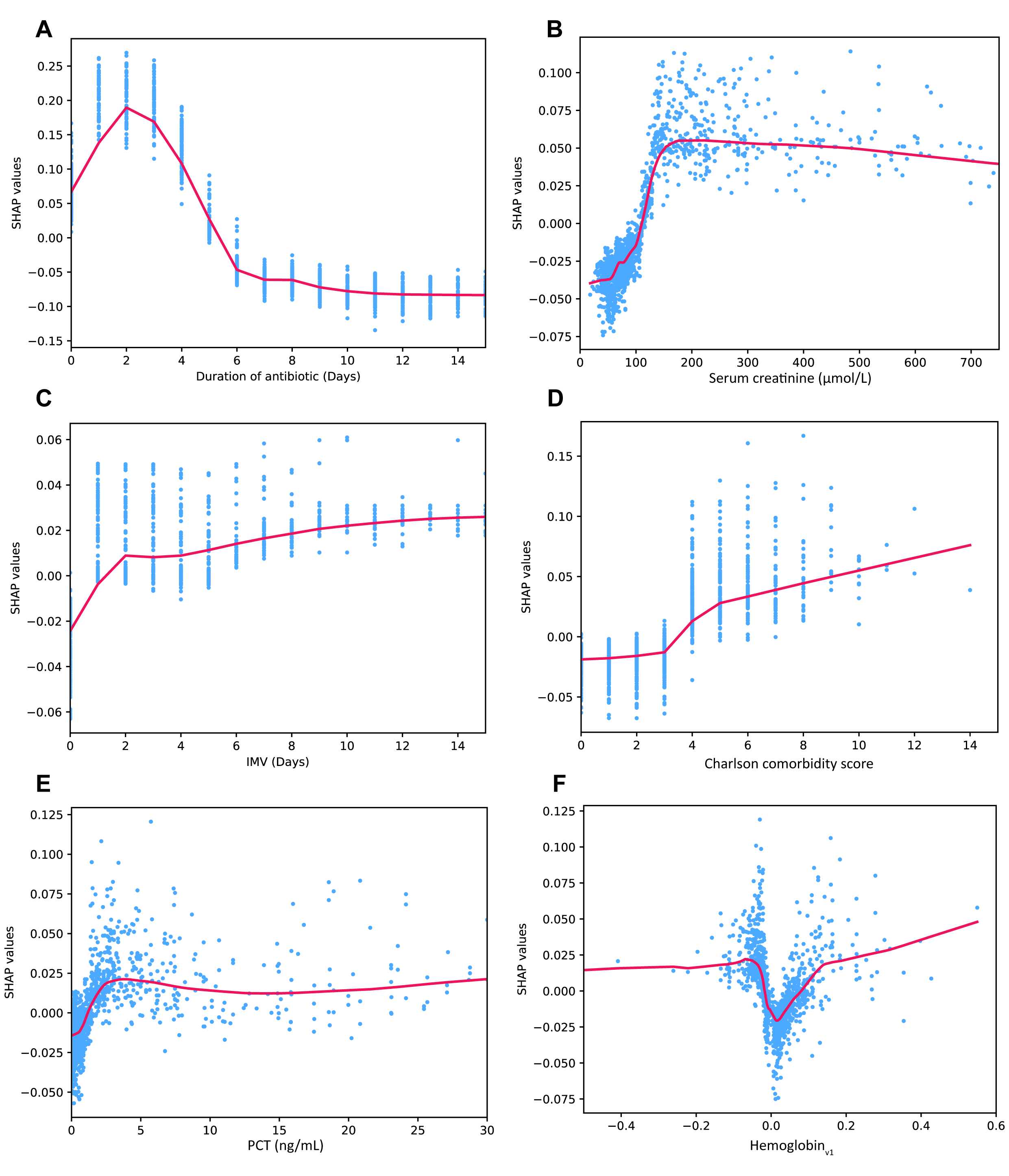
**

## Figure S7. Impact of variable changes on 28-day mortality in patients with ICU-BSIs.

A. Antibiotic duration: Reduced mortality risk after >2 days. B. Serum creatinine: Increased mortality risk at > 110 µm/L. C. IMV duration: Increased mortality risk after > 1 day. D. CCI: Increased mortality risk at > 3. E. PCT: Increased mortality risk at > 0.1 ng/ml. F. Hemoglobin: Stable levels associated with lowest risk; Fluctuations (increase or decrease) increase risk.

**
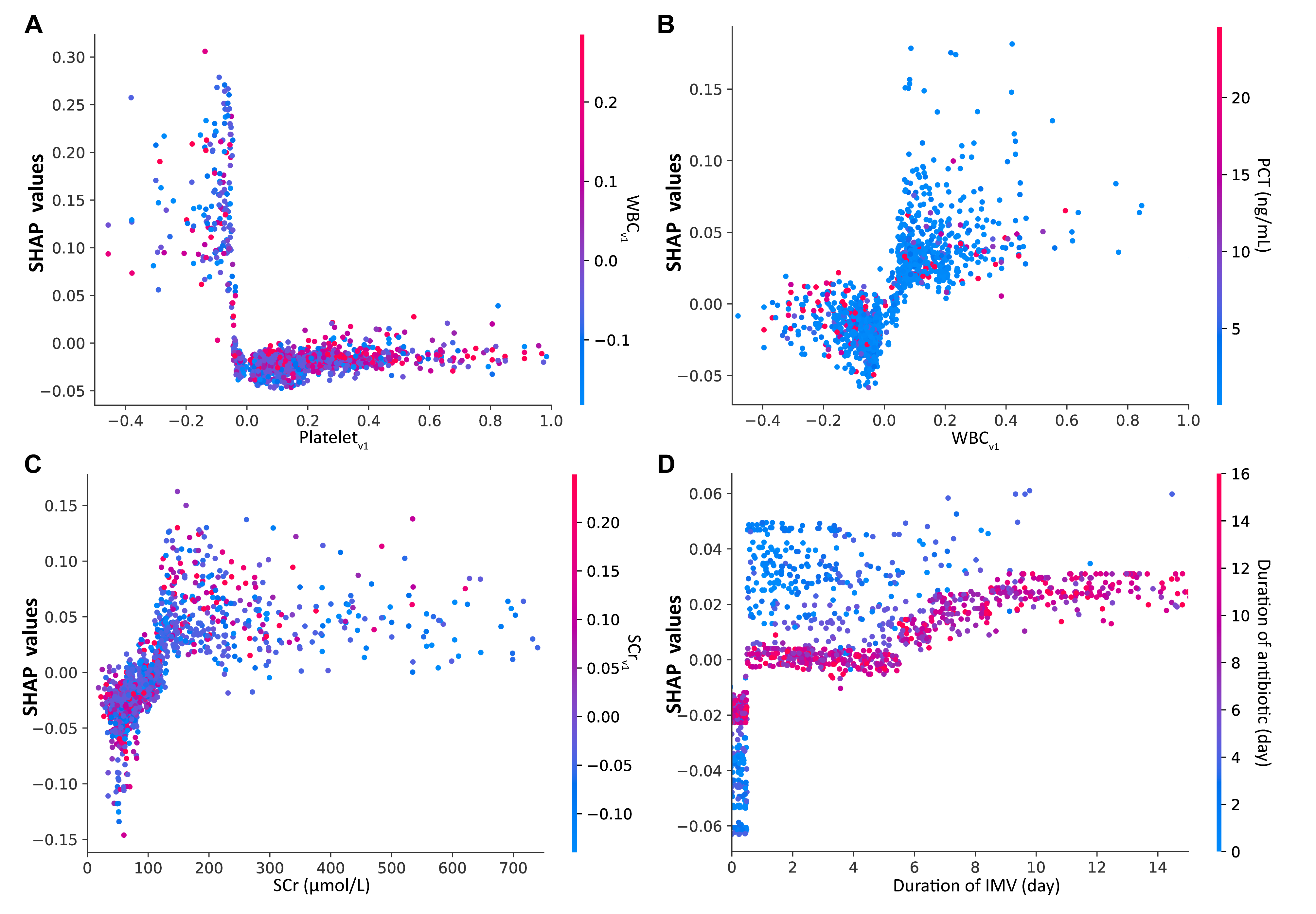
**

## Figure S8. Interactions among common clinical variables and 28-day mortality.

1. Platelet change rate x white blood cell change rate. B. White blood cell change rate x procalcitonin (PCT). C. Serum creatinine x creatinine change rate. D. Antibiotic duration x invasive mechanical ventilation (IMV).


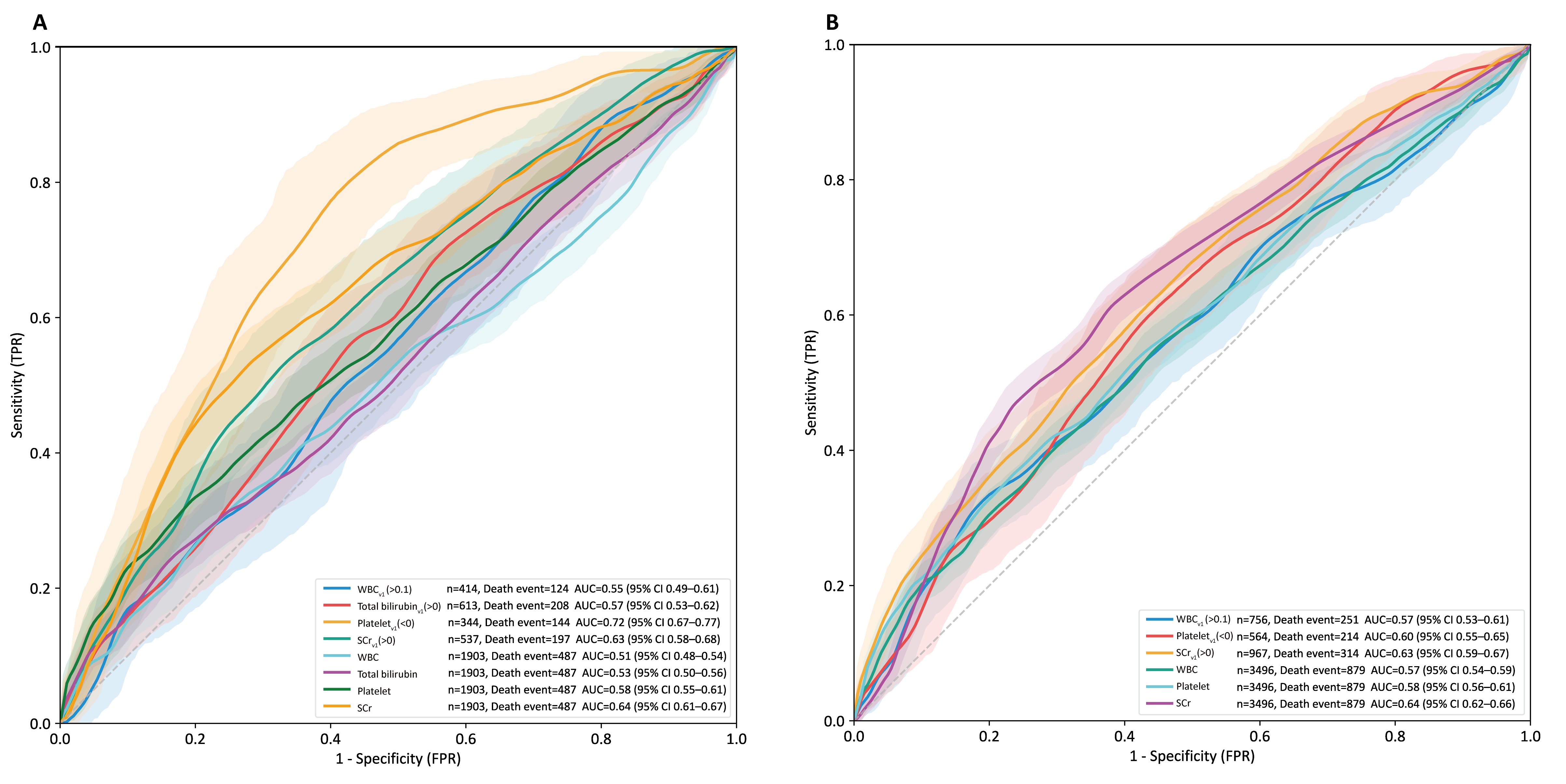


## Figure S9. AUROC curve of different variables in the AMU(A) and MIMIC-IV(B) dataset.
